# Supplementary material for: Evidence for Electron Transfer from the Bidirectional Hydrogenase to the Photosynthetic Complex I (NDH-1) in the Cyanobacterium Synechocystis sp. PCC 6803
Source: Microorganisms. 2022 Aug 10;10(8):1617. doi: 10.3390/microorganisms10081617 (PMC9414918; doi:10.3390/microorganisms10081617)
Supplement: Supplementary file 1 [file microorganisms-10-01617-s001.zip › microorganisms-1817042-supplementary.pdf]

Supplementary Material

Evidence for electron transfer from the bidirectional hydrogenase to the photosynthetic complex I (NDH-1) in the cyanobacterium *Synechocystis* sp. PCC 6803

Jens Appel[1,3], Sean Craig[2], Marius Theune[1,3], Vanessa Hüren[3], Sven Künzel[4], Björn Forberich[3], Samantha Bryan[2], Kirstin Gutekunst[1,3]

[1]Department of Molecular Plant Physiology, Bioenergetics in Photoautotrophs, University of Kassel, D-34132 Kassel, Germany

[2]BBSRC/EPSRC Synthetic Biology Research Centre, The Biodiscovery Institute, University of Nottingham, Nottingham, NG7 2RD, UK

[3]Department of Biology, Botanical Institute, Christian-Albrechts-University, D-24118 Kiel, Germany

[4]Max-Plank Institute for Evolutionary Biology, August-Thienemann-Straße 2, D-24306 Plön, Germany

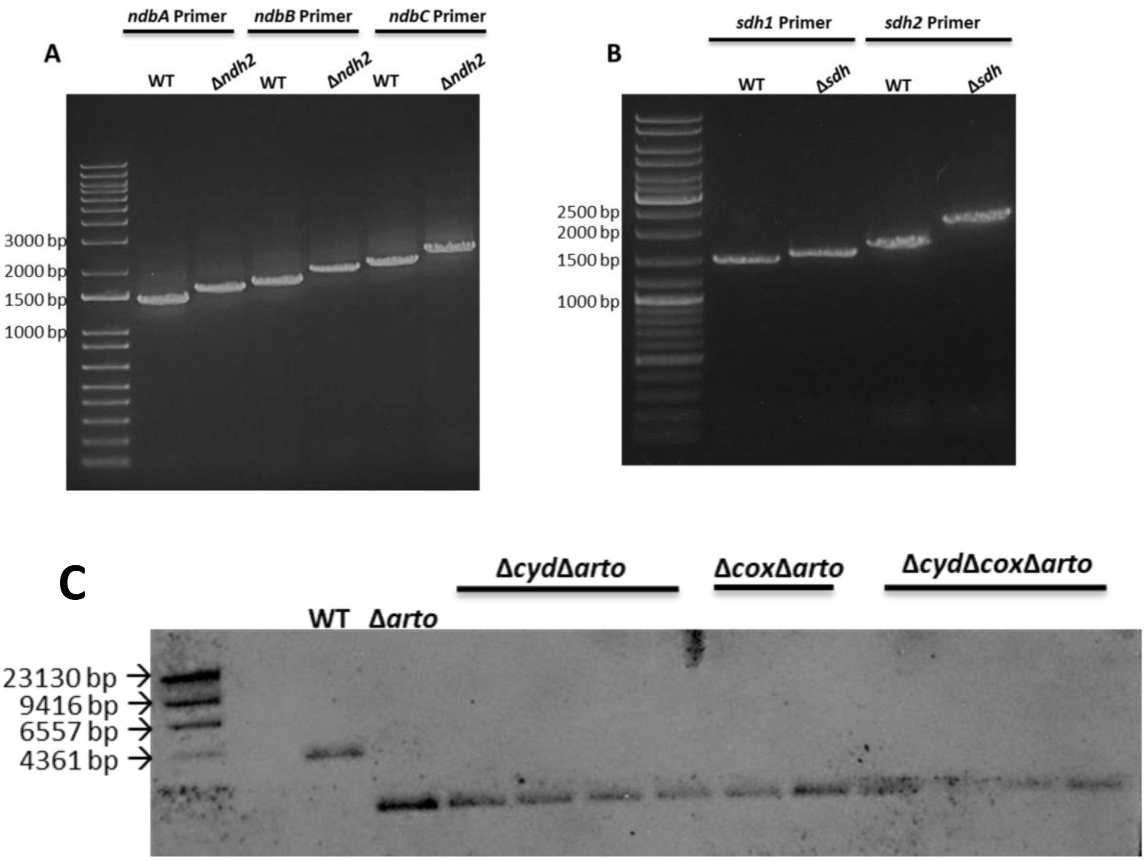

Figure S1. Agarose gels of PCR and southern hybridization to test segregation of the mutants. A) the deletion mutant of all the different type 2 dehydrogenase genes, B) the deletion mutant of the two *sdhB* genes, C) deletion strain of all the respiratory terminal oxidases (Δ*cyd*Δ*cox*Δ*arto*) called Δ*ox* in this study.

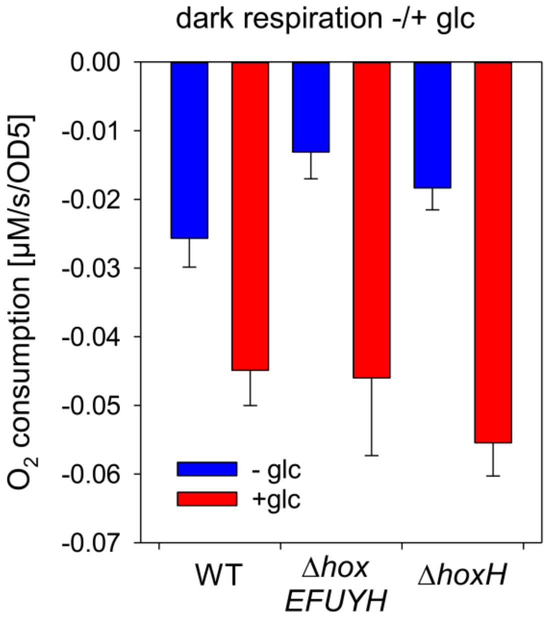

Figure S2. Dark oxygen consumption before and after addition of 10 mM glucose. The respiratory activity is given as µM O_2 consumed per second and at a density of OD_{750} = 5. Error bars show the standard deviation.

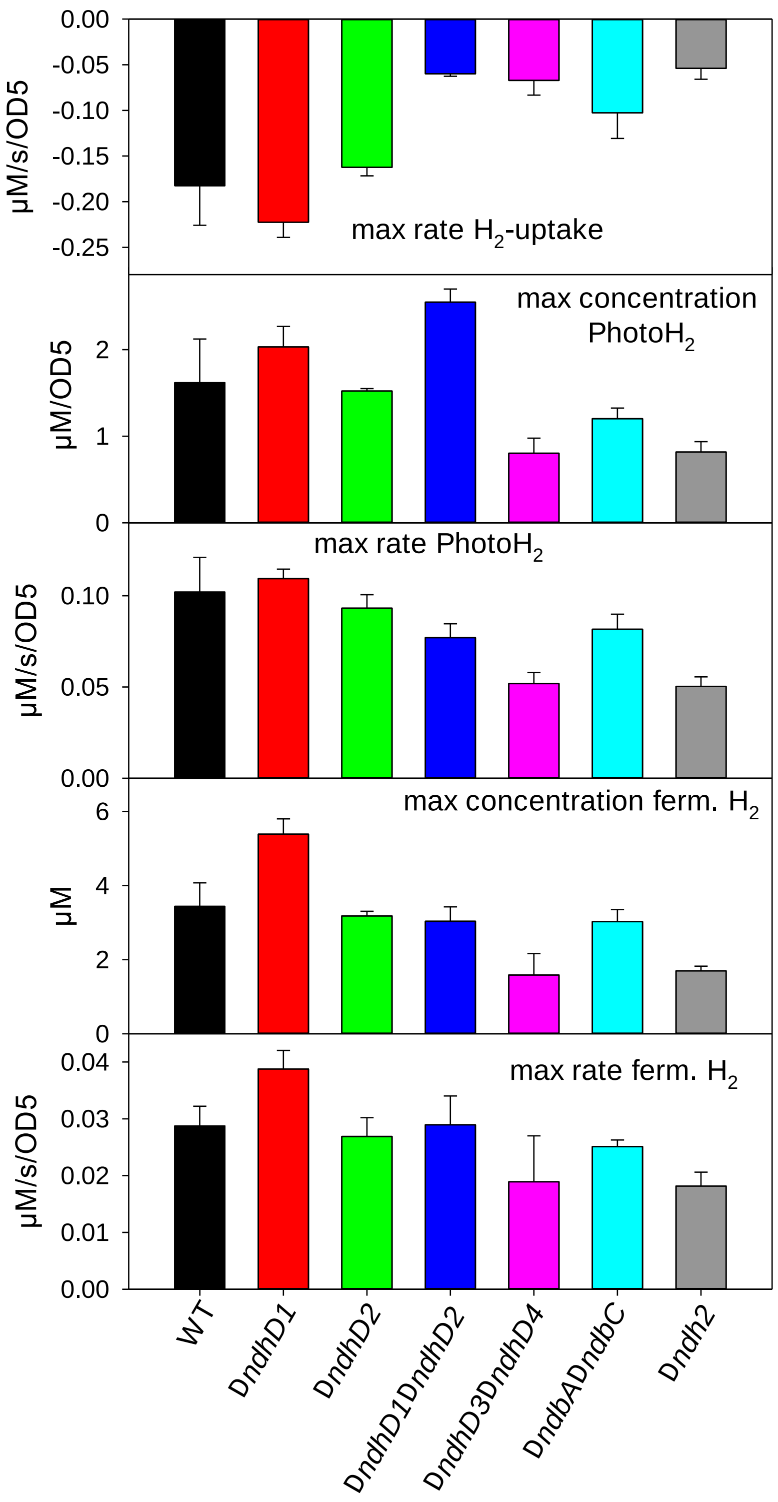

Figure S3. Analysis of the photoH_2 measurements. Experiments were performed as shown in Figure 2 for at least three different cultures of the same strain. During the course of the experiment the maximal rates of the different phases (fermentative H_2 production, PhotoH_2 production and H_2-uptake) were determined as well as the maximal concentrations of produced fermentative H_2 and PhotoH_2. Rates are given as µM H_2 produced or consumed per second and at a density of OD_{750} = 5. The maximum of photoH_2 production is also given for the sane density. Please note that fermentative H_2 production is limited by thermodynamics and thus is independent of cell density while the amount of photoH_2 depends on the cell density, chlorophyll concentration and shading and is given as per cell density. Error bars show the standard deviation.

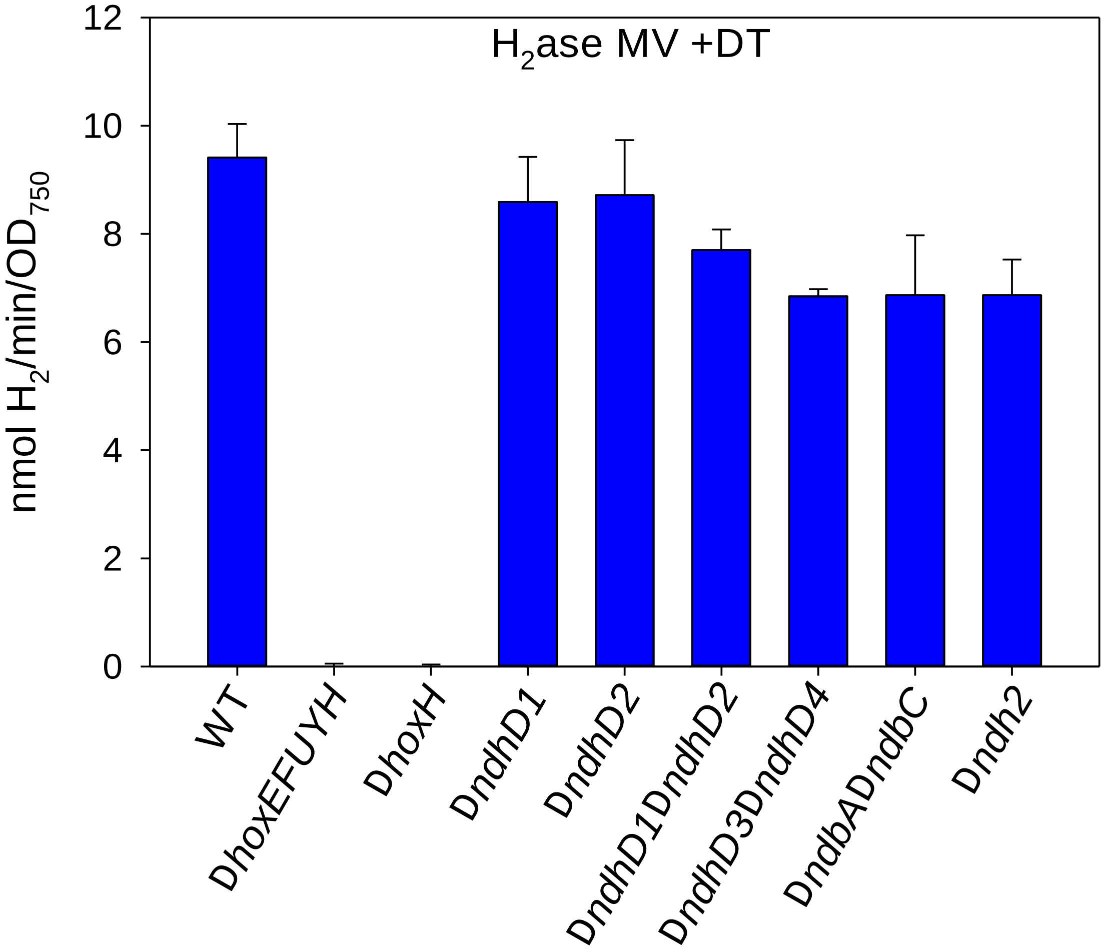

Figure S4. Hydrogenase activity as measured by the addition of 5 mM methylviologen and 10 mM dithionite to cell suspensions. The activity is directly proportional to the amount of hydrogenase present in the cells [14]. Error bars show the standard deviation of at least three independent biological replicates.

A

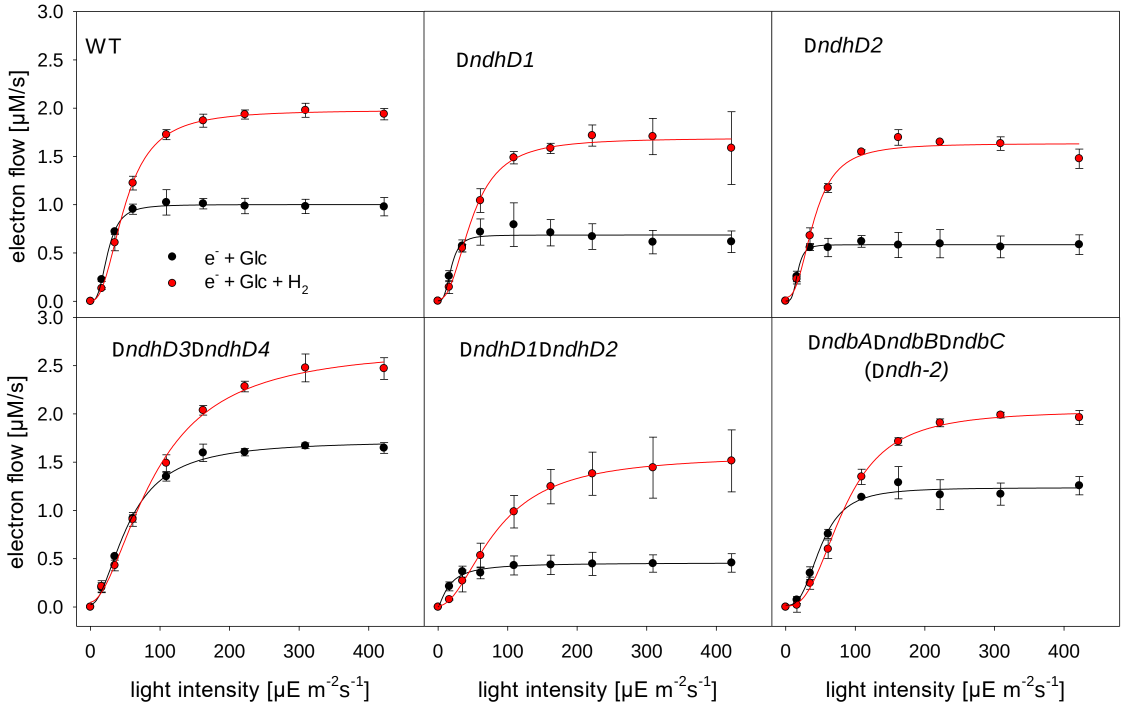

B

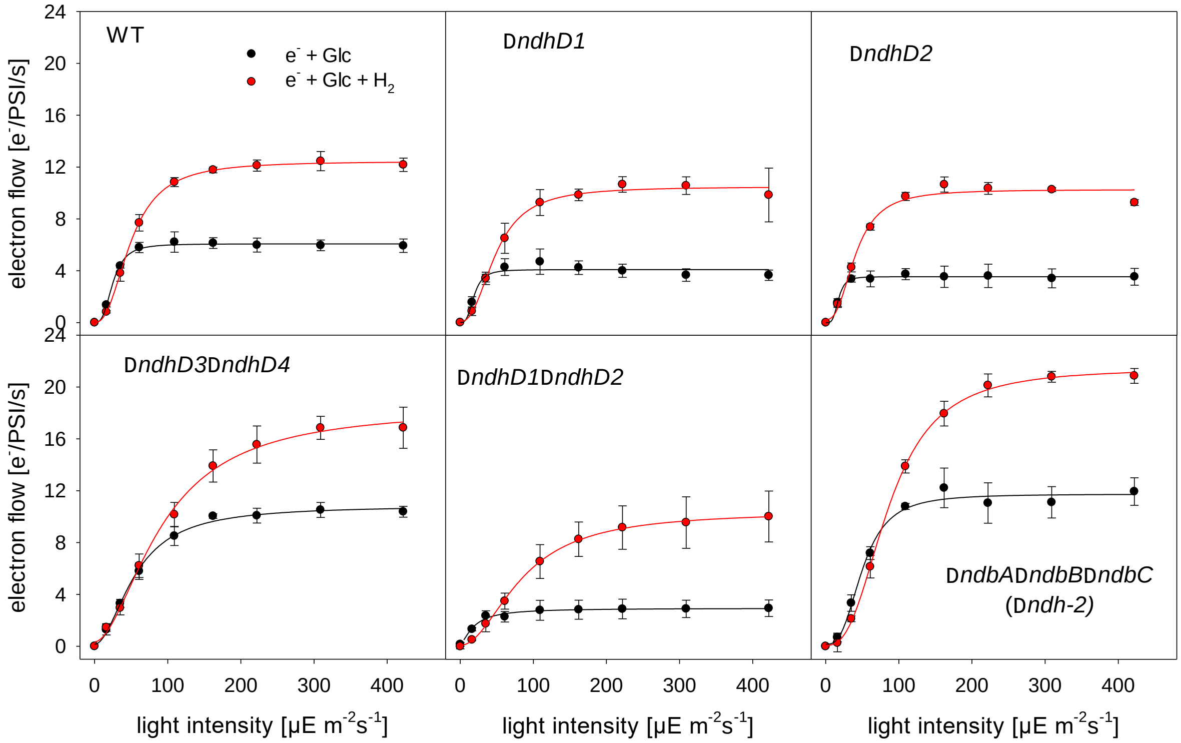

Figure S5. Electron flow through PSI as calculated from the DIRK measurements in the absence (black symbols and curves) and presence (red symbols and lines) of hydrogen. In (A) the values are given as µM e^-/s and in (B) as e^-/PSI/s. The difference of both curves in (A) is blotted in Figure 4. In A the rates are given as µM electrons/s at a cell density of OD_{750} = 5.7. The error bars In (A) and (B) indicate the standard deviation of at least three independent cultures.

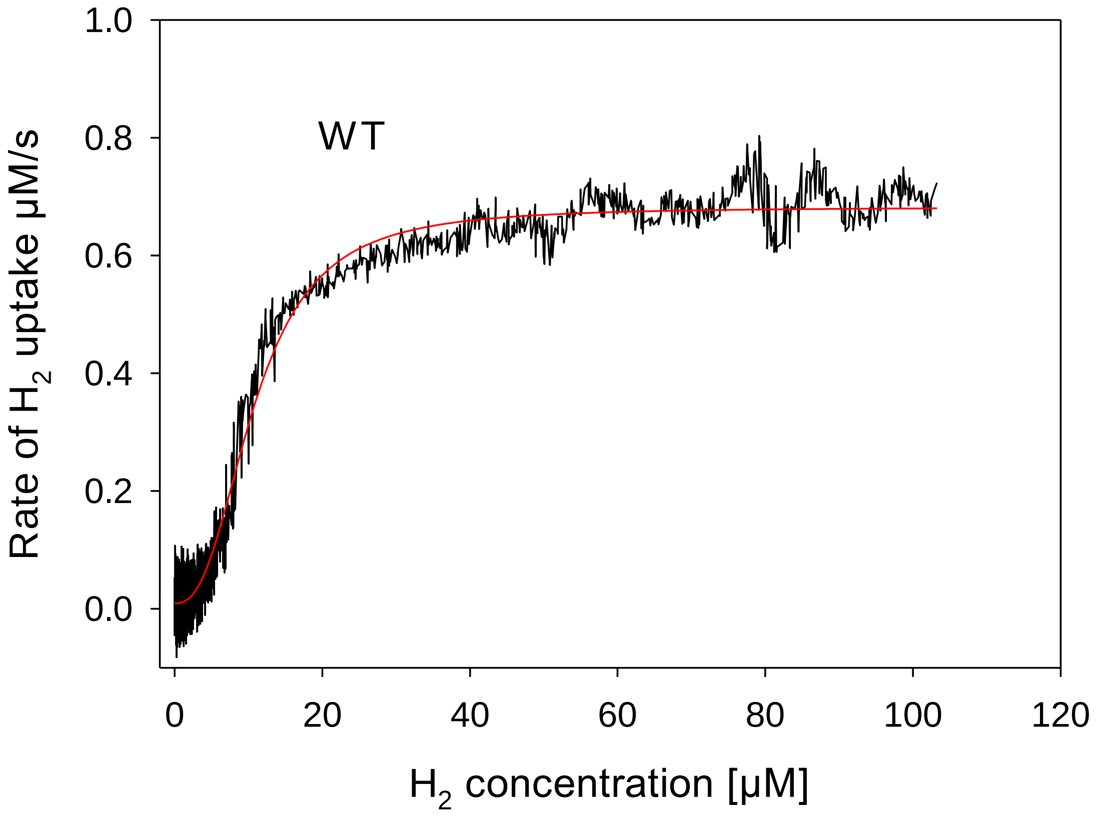

Figure S6. Hydrogen uptake rates plotted against actual H_2 concentration. H_2 uptake was followed in the light until completion. From the concentration curve as measured by the electrode the rate was calculated and plotted above against the H_2 concentration. The Michaelis-Menten blot thus gained was fitted with a Hill-equation (red line).

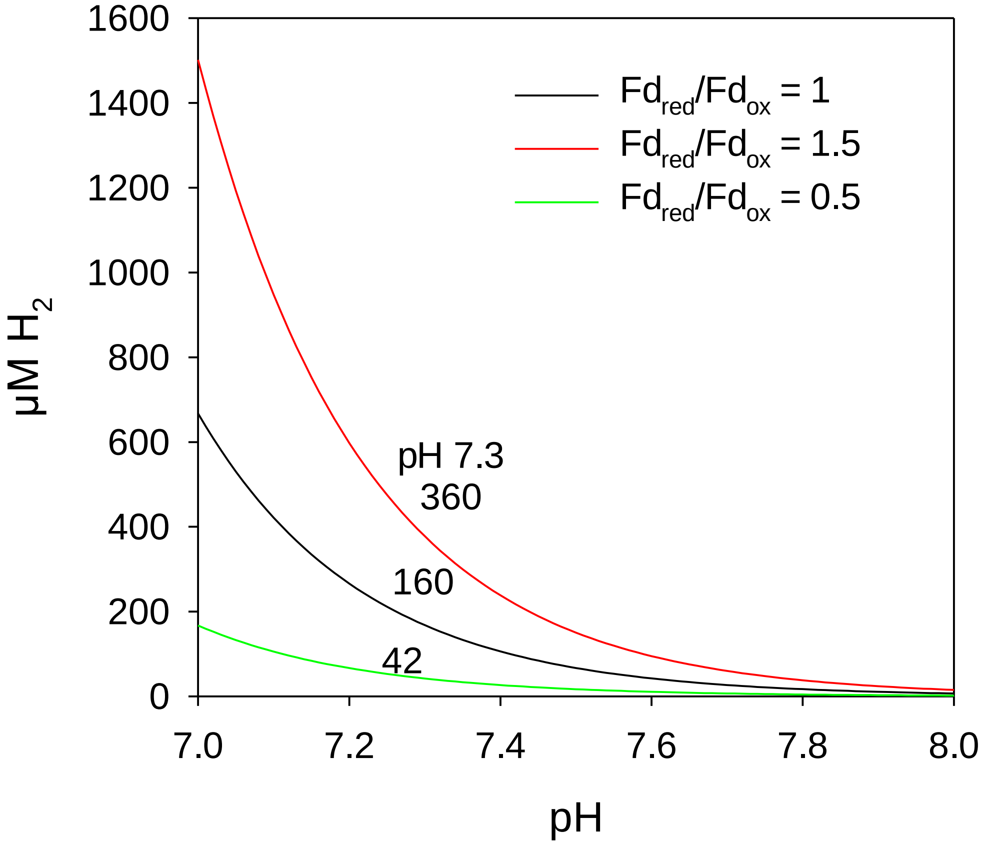

Figure S7. H_2 concentration curves in equilibrium with a specific Fd_{red}/Fd_{ox} ratio plotted against pH. To calculate the H_2 concentration that is in equilibrium with a specific ratio of reduced to oxidized ferredoxin 1 the Nernst equation was used and the data was plotted against the pH. At an intracellular pH of 7.3 the minimal concentration of hydrogen that could be reached for e.g. a ratio of Fd_{red}/Fd_{ox} of 0.5 is 42 μM.

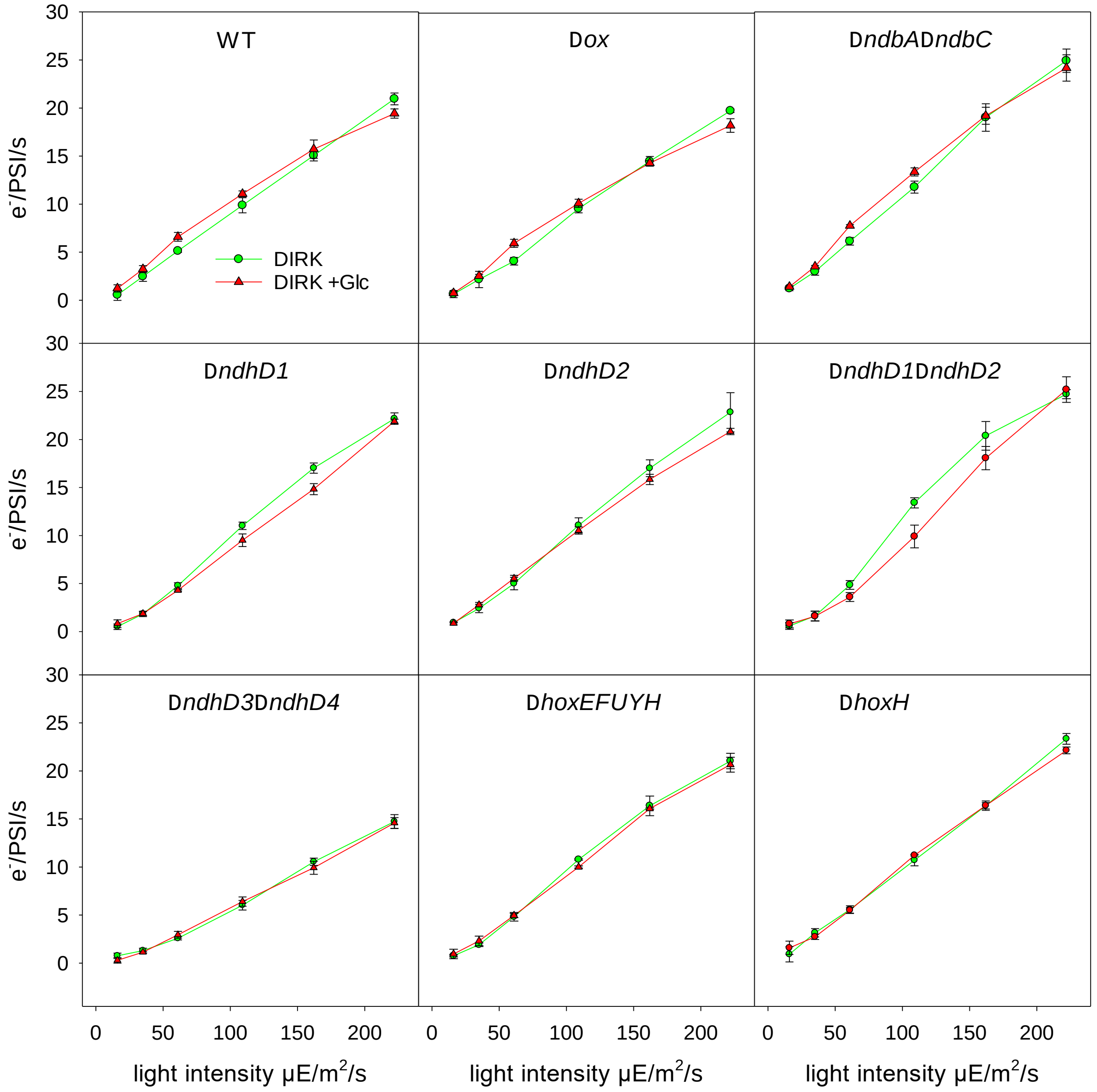

Figure S8. Electron flow through PSI as measured by DIRK before (green) and after (red) glucose addition. Only wild type cells, the mutant without terminal respiratory oxidases (Δ*ox*) and the mutant lacking the type 2 dehydrogenases (Δ*ndbA*Δ*ndbC*) show a slightly increased electron flow after glucose addition in the range of their growth light intensity.

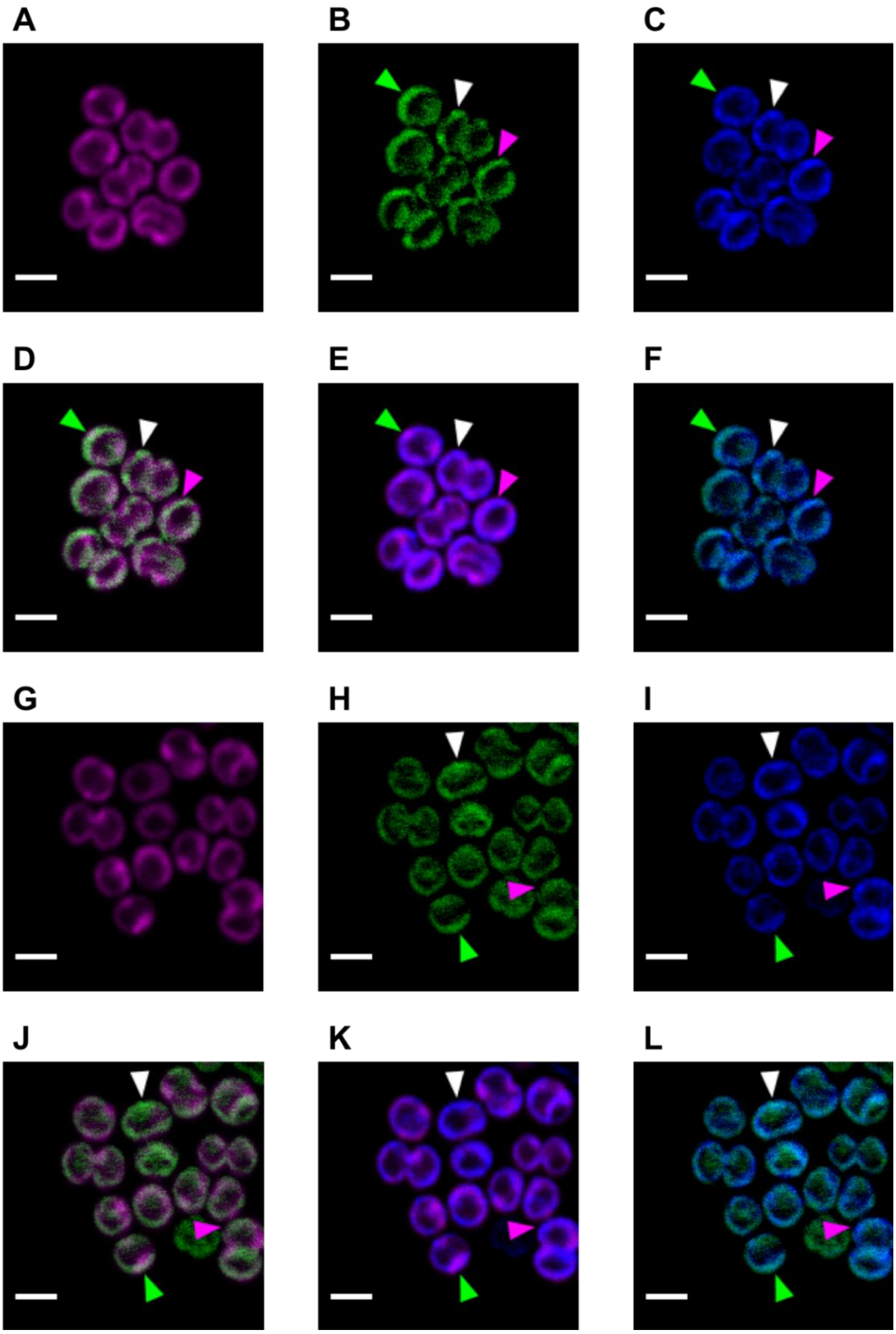

Figure S9. Distribution of Hox and NDH-1 in *Synechocystis* wild type cells grown photoautotrophically at 30 μmole photons m^{-2} s^{-1} and following anoxia. Confocal fluorescence micrographs for photoautotrophic (A-C) and anoxic (G-I) showing the chlorophyll fluorescence, GFP fluorescence and YFP fluorescence respectively. Fluorescence micrograph overlays for photoautotrophic (D-F) and anoxic conditions (J-L) showing the merge of chlorophyll/GFP, chlorophyll/YFP and GFP/YFP respectively. Scale bar represent 2 μm. (A-F) Cells grown a at 30 μmole photons m^{-2} s^{-1}. (G-L) Cells grown at 30 μmole photons m^{-2} s^{-1} with the addition of glucose oxidase and catalase. The chlorophyll/GFP fluorescent micrographs are overlaid with chlorophyll in magenta and GFP (Hox) in green (D & J). The chlorophyll/YFP fluorescent micrographs are overlaid with chlorophyll in red and YFP (NDH-1) in cyan. The Hox and NDH-1 fluorescent micrographs are overlaid with GFP in green and YFP in magenta. Green arrows highlight regions of Hox localisation. Magenta arrows highlight regions of NDH-1 localisation. White arrows highlight regions of Hox and NDH-1 co-localisation.

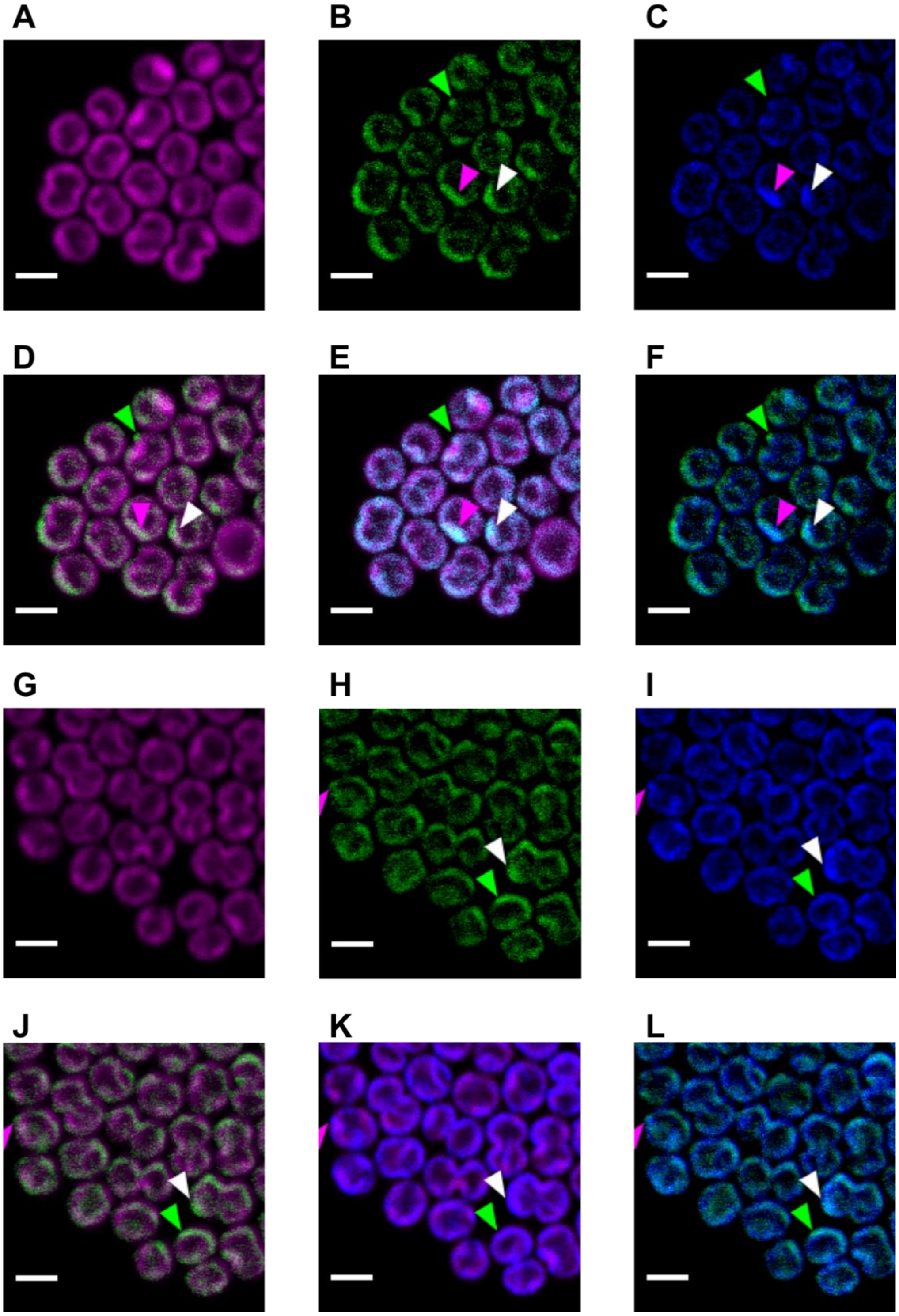

Figure S10. Distribution of Hox and NDH-1 in *Synechocystis* Δ*ndhD1*Δ*ndhD2* grown photoautotrophically at 30 μmole photons m^{-2} s^{-1} and following anoxia (A-L). Confocal fluorescence micrographs for photoautotrophic (A-C) and anoxic (G-I) showing chlorophyll fluorescence, GFP fluorescence and YFP fluorescence respectively. Fluorescence micrograph overlays for photoautrophic growth (D-F) and anoxic conditions (J-L) showing the merge of chlorophyll/GFP, chlorophyll/YFP and GFP/YFP respectively. Scale bar represents 2 μm. (A-F) Cells grown a at 30 μmole photons m^{-2} s^{-1}. (G-L) Cells grown at 30 μmole photons m^{-2} s^{-1} with the addition of glucose oxidase and catalase. The chlorophyll/GFP fluorescent micrographs are overlaid with chlorophyll in magenta and GFP (hox) in green (D & J). The chlorophyll/YFP fluorescent micrographs are overlaid with chlorophyll in red and YFP(NDH-1) in cyan. The Hox and NDH-1 fluorescent micrographs are overlaid with GFP in green and YFP in magenta. Green arrows highlight regions of Hox localisation. Magenta arrows highlight regions of NDH-1 localisation. White arrows highlight regions of Hox and NDH-1 colocalization.

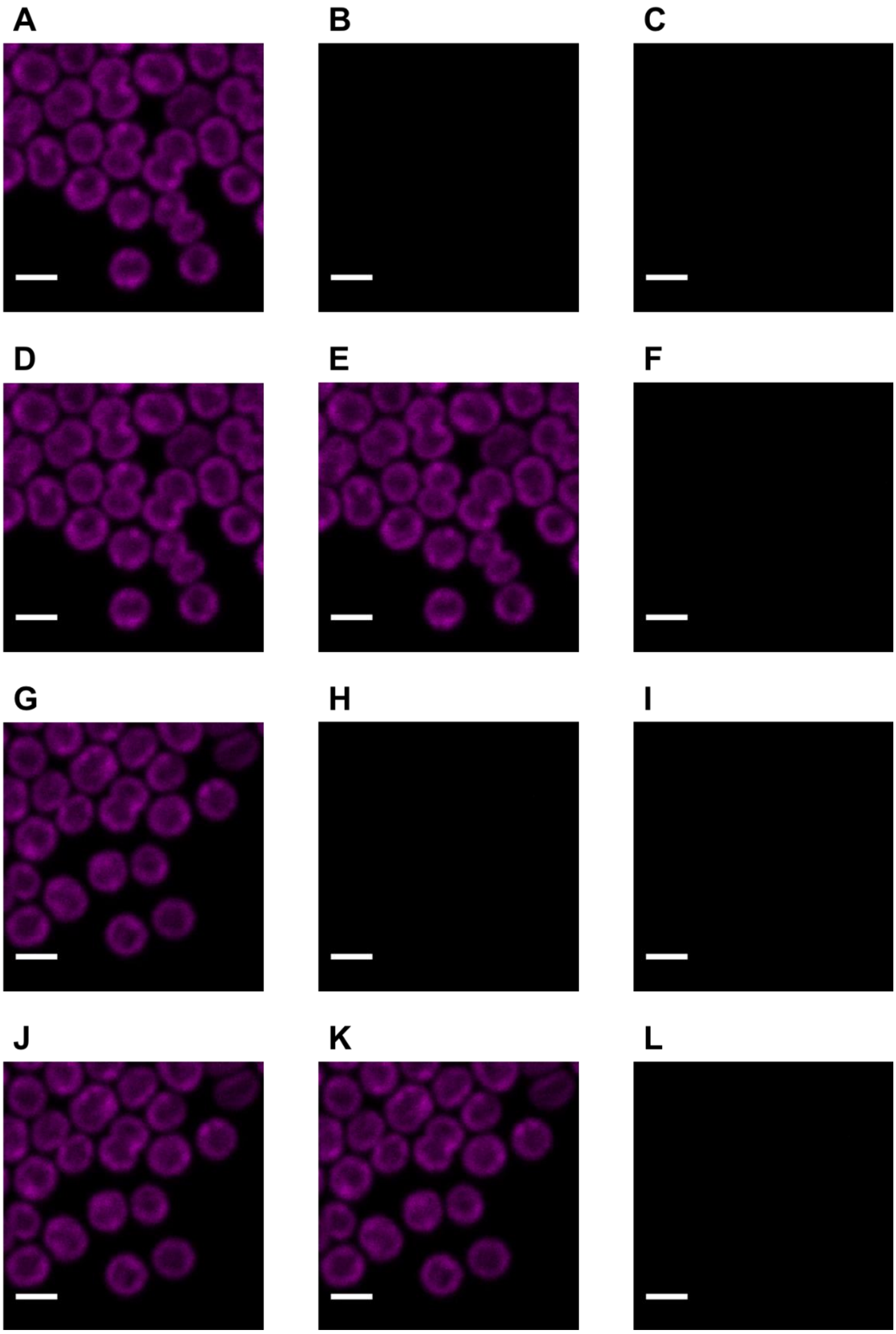

Figure S11. Assessment of autofluorescence in *Synechocystis* wild type cells grown photoautotrophically at 30 µE/m^2/s and following anoxia. Confocal fluorescence micrographs for photoautotrophic (A-C) and anoxic (G-I) conditions showing photosynthetic pigment fluorescence, GF-fluorescence and YFP-fluorescence, respectively. Fluorescence micrograph overlay for photoautotrophic (D-F) and anoxic conditions (J-L) showing the merge of photosynthetic pigments/GFP, photosynthetic pigments/YFP and GFP/YFP, respectively. Scale bar represents 2 µm. (A-F) cells grown at 30 µE/m^2/s. (G-L) cells grown at 30 µE/m^2/s with the addition of glucose, glucose oxidase and catalase.

Table S1: Primers used in this study for plasmid construction and PCR screening.

**Primer name**	**sequence**	**Fragment amplified**	**construct**
coxout1	CTATAGGGCGAATTGGGTACATTACGGTTAAAGCAGGAT	upstream recombination-site	Deletion of *ctaDI* (*slr1137*)
coxin1	AGAGATTTATCTAATTTCTTTTTTCGTCGACGATTCTCAGCGGCAATAGTCATAAA		
Em1	GTCGACGAAAAAAGAAATTAGATAAA	Em-cassette	
Em2	GTCGACTTACTTATTAAATAATTTATAGC		
coxin2	GCTATAAATTATTTAATAAGTAAGTCGACGATGCGGCAGGAAGTTAGTTT	downstream recombination-site	
coxout2	AGGGAACAAAAGCTGGAGCTGGCATCACACTGCGATAAAT		
cydout1	CTATAGGGCGAATTGGGTACAGAAGGAGTTTACGATCGCCAA	upstream recombination site	Deletion of *cydA* and *cydB* *(slr1379* and *slr1380)*
cydin1	TTGGCACCCAGCCTGCGCGATTACTCAAAAAATCCTGCATCTGTAA		
Sp-KG	TCGCGCAGGCTGGGTGCCAA	Sp-cassette	
Sp-rev	GCCCTCGCTAGATTTTAATGCGGAT		
cydin2	ATCCGCATTAAAATCTAGCGAGGGCAAAATTGTCACCGACTAGGGAGTT	downstream recombination site	
cydout2	AGGGAACAAAAGCTGGAGCTTGCAACGGGTCAGCATCCAATTT		
ARTOout1	CTATAGGGCGAATTGGGTACCTGGATCAGCTAATTACCCTAATTAGTA	upstream recombination site	deletion of *ctaDII* and *ctaEII* of the alternative respiratory terminal oxidase (*slr2082*, and *slr2083)*
ARTOin1Gm	GGTTCGTGCCTTCATCCGTCGACAGCGGGAAAGGGCAGTGCTTGTTT		
Gm1	GTCGACGGATGAAGGCACGAACC	Gm-cassette	
Gm2	GTCGACCGAATTGTTAGGTGGCG		
ARTOin2Gm	CGCCACCTAACAATTCGGTCGACCCAGCGAATTAAATCTTTGGCAT	downstream recombination site	
ARTOout2	AGGGAACAAAAGCTGGAGCTCTAGAATTCCACAGTCATAGGCAA		
ndbAout1	CTATAGGGCGAATTGGGTACAATATTTTCGCCGTTGCTATGAA	upstream recombination site	deletion of *ndbA* (*slr0851)*
ndbAin1	GGTTCGTGCCTTCATCCGTCGACGCATGGTCCTCCAACACCACTTT		
Gm1	GTCGACGGATGAAGGCACGAACC	Gm-cassette	
Gm2	GTCGACCGAATTGTTAGGTGGCG		
ndbAin2	CGCCACCTAACAATTCGGTCGACTAATATATTTGTCCTGGGGGATTT	downstream recombination site	
ndbAout2	AGGGAACAAAAGCTGGAGCTAGCTATGGTGGGGTTTACCGAA		
ndbBout1	CTATAGGGCGAATTGGGTACCCACCAAAAGGCGATCGCCACTTA	upstream recombination site	deletion of *ndbB* (*slr1743)*
ndbBin1	TCAATAATATCGAATTCCTGCAGCCGTGGTCGAGCGTCCGTCATAAT		
Cm1	CTGCAGGAATTCGATATTATTG	Cm-cassette	
Cm2	AAGCTTGATGGCGGCACCTCGCT		
ndbBin2	AGCGAGGTGCCGCCATCAAGCTTAAAAATGAACCTTCCTGAGGGAAA	downstream recombination site	
ndbBout2	AGGGAACAAAAGCTGGAGCTATGGGGGTGGTAATAGGCCATT		
ndbCout1	CTATAGGGCGAATTGGGTACAATCACCGCCGCCCAGGTTCAAT		deletion of *ndbC* (*sll1484)*

ndbCin1	TTGGCACCCAGCCTGCGCGAAAAGTGGGGCCAATTTCCTGGAAA	upstream recombination site	
Sp-KG	TCGCGCAGGCTGGGTGCCAA	Sp-cassette	
Sp-rev	GCCCTCGCTAGATTTTAATGCGGAT		
ndbCin2	ATCCGCATTAAAATCTAGCGAGGGCACCGGAAAGGGAAAGGGCTCCTT	downstream recombination site	
ndbCout2	AGGGAACAAAAGCTGGAGCTGGACAATGATGGGATGGAGGGTAT		
sdhB1out1	CTATAGGGCGAATTGGGTACGACAGTTCTGCTTCCGGTCAA	upstream recombination site	deletion of *sdhB1* (*sll1625*)
sdhB1in1	GGTTCGTGCCTTCATCCGTCGACATTTTGCAAACAATTTCCATGGTA		
Gm1	GTCGACGGATGAAGGCACGAACC	Gm-cassette	
Gm2	GTCGACCGAATTGTTAGGTGGCG		
sdhB1in2	CGCCACCTAACAATTCGGTCGACTTCGTTTATTTGACTTGATGGAT	downstream recombination site	
sdhB1out2	AGGGAACAAAAGCTGGAGCTTGTGACCTGGCAATTTTGATGT		
sdhB2out1	CTATAGGGCGAATTGGGTACGAGAGTTTGGCCCAAAAGTTGA	upstream recombination site	deletion of *sdhB2* (*sll0823)*
sdhB2in1	TCAATAATATCGAATTCCTGCAGCTGTTGCGGTTTTTGGCGCAA		
Cm1	CTGCAGGAATTCGATATTATTG	Cm-cassette	
Cm2	AAGCTTGATGGCGGCACCTCGCT		
sdhB2in2	AGCGAGGTGCCGCCATCAAGCTTGCGGAAAAACTTGCCAATTTCT	downstream recombination site	
sdhB2out2	AGGGAACAAAAGCTGGAGCTTAACCGTTCCAATCGACTTT		
HoxF_Up_F	ACTAGTGAATTCGCGGCCGCCTGCAAATGTACAAAATCCCACC	upstream recombination site	construct for GFP-labelling of HoxF
HoxF_Up_R	CGGGCCCGGCAGGACTTTGAGTAATTCTTCATATTC		
HoxF_GFP_F	ATTACTCAAAGTCCTGCCGGGCCCGGAGCTG	GFP and apramycin cassette	
HoxF_GFP_R	ATAAATTACCGAAATTCCGGGGATCCGTCGACCTG		
HoxF_Down_F	GATCCCCGGAATTTCGGTAATTTATCCACTCAG	downstream recombination site	
HoxF_Down_R	CTATGCATCCAACGCGTTGGGAGCTCATGATTGCCTTCGGAAAAAAG		
HoxF_SC_F	TGTGGATTGGGGATGAGTGC	PCR screening	
HoxF_SC_R	TCCCAATGACCGGGTTTACG		
NdhM_Up_F	ACTAGTGAATTCGCGGCCGCCTGCACCCCAGCTTGGGCACTGT	upstream recombination site	construct for YFP-labelling of NdhM
NdhM_Up_R	GGGCCCGGCAGCGTTATCCAGCCAATATTTTCCTGCAGTTTC		
NdhM_YFP_F	TTGGCTGGATAACGCTGCCGGGCCCGGAGCT	YFP and spectinomycin cassette	
NdhM_YFP_R	AATTGATACCAAGTGTAGGCTGGAGCTGCTTCGAAGTTCCTATACTTTCTAGAGAATAGG		
NdhM_Down_F	CTCCAGCCTACACTTGGTATCAATTGACCATAACTGGAGGGAG	downstream recombination site	
NdhM_Down_R	CTATGCATCCAACGCGTTGGGAGCTAAGGGGCGGTCCAAGCCG		
NdhM_SC_F	TGGCTCGGATTTGGAGCATT	PCR screening	
NdhM_SC_R	GCACAGTGTTAAGCCTCCCT		
